# Supplementary material for: Association between oral microbiome and seven types of cancers in East Asian population: a two-sample Mendelian randomization analysis
Source: Front Mol Biosci. 2023 Nov 21;10:1327893. doi: 10.3389/fmolb.2023.1327893 (PMC10702768; doi:10.3389/fmolb.2023.1327893)
Supplement: Supplementary file 1 [file DataSheet1.ZIP › Supplementary Materials/MR plots of saliva/saliva═╝/Breast cancer/pheno.125_to_breast cancer_forest.pdf]

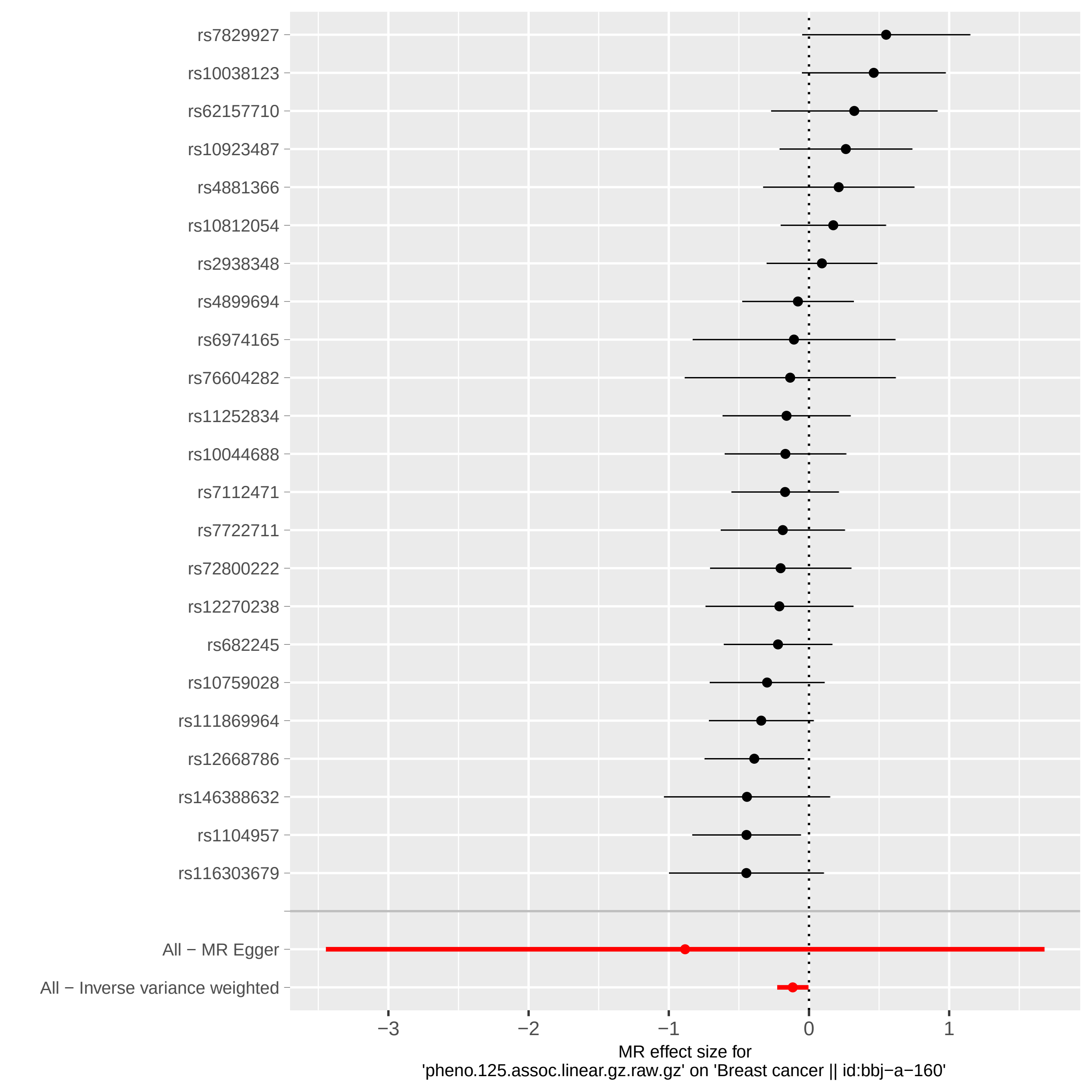
rs7829927
rs10038123
rs62157710
rs10923487
rs4881366
rs10812054
rs2938348
rs4899694
rs6974165
rs76604282
rs11252834
rs10044688
rs7112471
rs7722711
rs72800222
rs12270238
rs682245
rs10759028
rs111869964
rs12668786
rs146388632
rs1104957
rs116303679
All − MR Egger
All − Inverse variance weighted
−3
−2
−1
0
1
MR effect size for
'pheno.125.assoc.linear.gz.raw.gz' on 'Breast cancer || id:bbj−a−160'
